# Understanding Microbial Multi-Species Symbioses

**DOI:** 10.3389/fmicb.2016.00180

**Published:** 2016-02-18

**Authors:** Ines A. Aschenbrenner, Tomislav Cernava, Gabriele Berg, Martin Grube

**Affiliations:** ^1^Institute of Environmental Biotechnology, Graz University of TechnologyPetersgasse, Graz, Austria; ^2^Institute of Plant Sciences, University of GrazGraz, Austria; ^3^Austrian Centre of Industrial Biotechnology – Gesellschaft mit beschränkter HaftungGraz, Austria

**Keywords:** lichens, symbiosis, microbiome, *Alphaproteobacteria* host-associated bacteria

## Abstract

Lichens are commonly recognized as a symbiotic association of a fungus and a chlorophyll containing partner, either green algae or cyanobacteria, or both. The fungus provides a suitable habitat for the partner, which provides photosynthetically fixed carbon as energy source for the system. The evolutionary result of the self-sustaining partnership is a unique joint structure, the lichen thallus, which is indispensable for fungal sexual reproduction. The classical view of a dual symbiosis has been challenged by recent microbiome research, which revealed host-specific bacterial microbiomes. The recent results about bacterial associations with lichens symbioses corroborate their notion as a multi-species symbiosis. Multi-omics approaches have provided evidence for functional contribution by the bacterial microbiome to the entire lichen meta-organism while various abiotic and biotic factors can additionally influence the bacterial community structure. Results of current research also suggest that neighboring ecological niches influence the composition of the lichen bacterial microbiome. Specificity and functions are here reviewed based on these recent findings, converging to a holistic view of bacterial roles in lichens. Finally we propose that the lichen thallus has also evolved to function as a smart harvester of bacterial symbionts. We suggest that lichens represent an ideal model to study multi-species symbiosis, using the recently available omics tools and other cutting edge methods.

## Introduction

Twenty years after the theory of evolution by natural selection started to revolutionize biology, the German mycologist Anton de Bary introduced the term symbiosis to the broader scientific community as a living together of dissimilar organisms (de Bary, 1879). One of his prominent examples were lichens, even though the symbiotic nature – revealed earlier by Schwendener (1869) – was hardly accepted at that time. Scientific peers still considered them as an independent group of organisms with a unique morphology. Meanwhile every biology textbook includes lichens as an obligate association between a fungal (mycobiont) and a photosynthetic partner (photobiont), which can be either cyanobacteria and/or green algae (Nash, 2008). By this association, the photobiont’s production of energy via carbon dioxide fixation is enhanced by the sheltering structures of the exhabitant fungal partner. The joint structure, also known as the lichen thallus, is unique and one of the most complex vegetative structures in the entire fungal kingdom. The lichen thallus evolved as early as terrestrial plant life, as the first ancestors of lichens with characteristic morphology can be traced back to the Devonian 400 million years ago (Remy et al., 1994; Honegger et al., 2013). In this paper, we will show that lichens are not merely a partnership involving two unrelated organismal groups, but include a so far largely neglected bacterial component, which contributes to the biology of the holobiont. We will start with some general aspects of the lichen ecology and will then continue with an outline how modern analytical tools are used to understand lichens as a fascinating case of a multisymbiosis.

The successful fungal symbiosis, which comprises more than 18,000 named species of fungi is characterized by a poikilohydric lifestyle, which enables lichens to colonize almost all terrestrial environments, ranging from tropical to polar climatic zones, and coastal to high altitude habitats. In addition, lichens grow on the surface of almost every kind of substrate including bare soils, rocks, and plants, but they can be also found in freshwater streams and in marine intertidal zones (Nash, 2008), and various man-made material surfaces. The vegetative bodies vary in color, size (a few millimeters to meters) and growth forms, and some may persist for several 1000 of years (Denton and Karlén, 1973). The wide variety of lichen thallus structures, which are primarily determined by the fungal partner, can be roughly divided into three most common morphological types: crustose, foliose, and fruticose growth forms. Other types exist, but are less frequent (Grube and Hawksworth, 2007). Internally, the vegetative body is either homoiomerous (without stratification), where the mycobiont and photobiont are evenly distributed in the lichen thallus, or heteromerous (with stratification), where at least a fungal upper layer and an algal layer underneath can be distinguished. Crustose lichens are characterized by the attachment of the entire lower surface to the substrate, whereas foliose and fruticose lichens are only partially attached (Büdel and Scheidegger, 2008), and usually have a more or less dense lower fungal layer. Sexual reproduction of the fungal partner requires the development of the species-specific thallus with appropriate algae, since fungal fruit-bodies directly arise in the mature lichen thallus and often incorporate thallus structures. Nevertheless, lichens also evolved various means of asexual reproduction to disperse symbiotic partners together in diverse and specific joint propagules (Büdel and Scheidegger, 2008).

Even though the literature continues to report on antibacterial or antifungal compounds from lichens (reviewed in Boustie and Grube, 2005), the long-lived thalli provide interesting microhabitats for other eukaryotic and prokaryotic (both bacteria and archaea) microorganisms (Lawrey and Diederich, 2003; Grube and Berg, 2009; Bjelland et al., 2011; Bates et al., 2012). In previous years attention was increasingly paid to lichen-associated bacteria that were not recognized as being an integral part of the symbiosis.

In this review we discuss recent literature on lichen-associated microbiota with focus on diversity, functions, dispersal, habitat specificity, and inter-microbiome relations of the *Lobaria pulmonaria*-associated bacterial community and conclude with an outline to promote a holistic view on lichen-bacteria interactions. In the first part we review historic aspects and then discuss recent results to develop a more holistic lichen model.

## Unraveling the Lichen-Associated Microbiome – then and Now

Bacteria associated with lichens were initially mentioned in the first half of the 20th century (Uphof, 1925; Henkel and Yuzhakova, 1936; Iskina, 1938). During these early studies various bacterial genera were reported to be associated with lichens such as *Azotobacter, Pseudomonas* (*Gammaproteobacteria*), *Beijerinckia* (*Alphaproteobacteria*), and the *Firmicutes* genera *Bacillus* and *Clostridium* (Iskina, 1938; Panosyan and Nikogosyan, 1966; Henkel and Plotnikova, 1973). At that time descriptions of bacteria underlay solely phenotypical and physiological characterizations indicating a possible role in nitrogen fixation for some of these bacteria. Nevertheless, Lenova and Blum (1983) already estimated that millions of bacterial cells per gram could colonize a lichen thallus. Several decades passed before the first molecular analyses started using bacterial isolates, (e.g., González et al., 2005; Cardinale et al., 2006, Liba et al., 2006, or Selbmann et al., 2009). While González et al. (2005) only focused on culturable *Actinomycetes* (with *Micromonospora* and *Streptomyces* as predominant genera) of various lichen species from tropical and cold areas, Cardinale et al. (2006) attempted to describe the overall bacterial community composition associated with seven different lichen species from temperate habitats. The latter enabled the identification of several genera affiliated to *Firmicutes*, *Actinobacteria*, and *Proteobacteria*, highlighting *Paenibacillus*, and *Burkholderia* to be ubiquitous genera in lichens. However, culture-dependent methods capture only 0.001–15% of the bacterial diversity in environmental samples (Amann et al., 1995), whereas the majority remains unobserved (Rappé and Giovannoni, 2003). To overcome the limitations of selective bacterial isolation from environmental samples and to obtain a more unbiased and less restricted view on the microbial communities, new techniques were employed to complement the traditional methods.

First culture-independent investigations on lichen-associated microbiota were assessed with different fingerprinting methods (Cardinale et al., 2006; Grube et al., 2009; Bjelland et al., 2011; Mushegian et al., 2011; Cardinale et al., 2012a) and molecular cloning approaches (Hodkinson and Lutzoni, 2009). Such techniques (e.g., DGGE: Muyzer and Smalla, 1998; T-RFLP: Liu et al., 1997; SSCP: Schwieger and Tebbe, 1998) were used to generate microbial community profiles by amplifying genetic markers (e.g., 16S ribosomal DNA) with universal primers. Based on sequence or length polymorphisms PCR products are separated and the degree of sample similarity according to the specific band patterns can be characterized (Smalla et al., 2007). Although many samples can be analyzed in parallel and their profiles can be compared with each other easily, the identification of the bacterial community members in detail is tedious and limited. Margulies et al. (2005) introduced a new time reduced and cost efficient technology to study community compositions and diversity of environmental samples in depth by large-scale high throughput sequencing. Bates et al. (2011) described lichen-associated bacteria for the first time based on this next generation pyrosequencing technology, followed by Grube et al. (2012), Hodkinson et al. (2012), and Aschenbrenner et al. (2014).

With the improvement of sequencing technologies and bioinformatics tools the focus in microbial ecology research shifted from the basic taxonomical descriptions to a more detailed and holistic view on microbial communities. Metagenomic, transcriptomic, and proteomic analyses can now shed light on the questions “Who is there?”, “What are they capable of?”, and “Who is actively doing what?” (Schneider et al., 2011; Aschenbrenner, 2015; Grube et al., 2015). To address these questions, the lung lichen *L. pulmonaria* (L.) Hoffm. was used as model system due to its relatively fast growth and other facilitative characteristics, e.g., epiphytic growth on tree bark and a low number of secondary metabolites, which could interfere with the conducted analyses. *L. pulmonaria* is characterized by a leaf-like structure (foliose lichen) and mainly found in old-growth forests with unpolluted air. Its sensitivity to air pollution can be employed for indirect evaluations of air quality and ecosystem integrity (Scheidegger and Werth, 2009). It harbors two photosynthetic partners, a phenomenon observed for approximately 4% of all described lichens (Honegger, 1991). However, only the green alga *Dictyochloropsis reticulata* forms a continuous layer, whereas cyanobacterial *Nostoc* strains are maintained in spaced, nodule-like internal compartments (cephalodia).

## Composition and Diversity of the Lichen-Associated Microbiome Driven by Various Abiotic and Biotic Factors

The amount of bacteria found on lichens is surprisingly high in relation to surfaces of higher plant foliage. While a leaf surface comprises only 10^5^ cells/cm^2^, some lichen species analyzed for bacterial abundance exceed this value dramatically (Saleem, 2015). For example, *Cladonia rangiferina*, is colonized by approximately 10^7^–10^8^ bacteria per gram of lichen thallus (Cardinale et al., 2008; Grube et al., 2009). Moreover, Alpha diversity indices (Shannon index) of bacterial communities were shown to vary between different lichens, e.g., from on average 4.5 (*Solorina crocea*) to 7.0 (*L. pulmonaria*) at a genetic distance of 3% among the microbial OTUs based on 16S rRNA gene sequence dissimilarity (Grube et al., 2012; Aschenbrenner et al., 2014).

*L. pulmonaria* is mainly colonized by *Alphaproteobacteria* with *Sphingomonadales* as the predominant order, followed by *Sphingobacteria*, *Actinobacteria*, and *Spartobacteria* (Aschenbrenner et al., 2014). Contrarily, shotgun sequencing-based studies suggested *Rhizobiales* as the main order within *Alphaproteobacteria* (Erlacher et al., 2015; Grube et al., 2015). These results were additionally confirmed with adapted visualizing techniques. Thereby, the predominance of *Alphaproteobacteria* and *Rhizobiales* on lichen surfaces were shown with a combined approach of fluorescence in situ hybridization (FISH) and confocal laser scanning microscopy (CLSM). Related to these findings, the lichen-associated *Rhizobiales* group (LAR1) was reported to be a lichen-specific lineage of *Alphaproteobacteria*, which can be found among many examined species (Hodkinson and Lutzoni, 2009; Bates et al., 2011; Hodkinson et al., 2012). However, this lineage could not be detected in *L. pulmonaria* (Aschenbrenner et al., 2014). The observed compositional differences within the same lichen species can be attributed to various reasons such as metagenomic sequencing approach (amplicon vs. shotgun sequencing), utilized databases, or activity of the bacteria in case of metatranscriptomic analysis (Aschenbrenner, 2015) since less than 10% of a microbial community is metabolically active at one time (Locey, 2010).

While the predominance of *Alphaproteobacteria* was also reported in other studies (Bates et al., 2011; Hodkinson et al., 2012), bacterial community composition in general differed among lichen species. These variations are supposed to be driven by various biotic and abiotic factors. Hodkinson et al. (2012) who thoroughly studied the bacterial communities associated with various lichen species comprising 24 mycobiont types with all photobiont combinations of different sampling locations (tropical and arctic regions) highlighted the photobiont type (chlorolichens vs. cyanolichens) and large-scale geography as the main driving forces.

Hodkinson et al. (2012) argued that the differences in community composition could be ascribed to both the availability of fixed nitrogen and the type of fixed carbon. Regarding the first one, bacteria associated with cyanolichens have access to fixed atmospheric nitrogen due to the cyanobacterial photobiont, whereas those of chlorolichens lack this benefit in nitrogen-restricted environments. According to that, chlorolichens would preferably enrich species capable of nitrogen fixation rather than cyanolichens. Another suggestion was that green algae release different types of fixed carbon (sugar alcohols: ribitol, erythritol, or sorbitol) than cyanobacteria (glucose; Elix and Stocker-Wörgötter, 2008), thereby shaping the bacterial community with respect to carbon utilization. Both explanations can only partly explain community differences based on taxonomic descriptions as bacteria can exchange and share genes encoding for certain functions via horizontal gene transfer. This agrees with Burke et al. (2011) who argued that ecological niches are colonized randomly by bacteria equipped with suitable functions rather than following bacterial taxonomy. The attempt to explain observed community compositions gets more complicated with regard to tripartite lichens as they carry both types of photobionts as it is the case in *L. pulmonaria*.

Species-specificity for bacterial communities associated with chlorolichens was already indicated in previous studies (Grube et al., 2009; Bates et al., 2011). Lichenized fungi are able to produce secondary metabolites, which are unique to lichens and comprise several 100 compounds which can be deposited on the extracellular surface of the fungal hyphae (Elix and Stocker-Wörgötter, 2008). As already suggested by Hodkinson et al. (2012) the considerable fraction of secondary metabolites with antimicrobial activities (Kosanić and Ranković, 2015) might cause a selective pressure on lichen-colonizing bacteria as well. However, as *L. pulmonaria* contains only low concentrations of lichen-specific substances like many other lichens of the suborder *Peltigerineae* (Beckett et al., 2003), secondary metabolites might play only a minor role in shaping the community structure of *Lobaria*-associated bacteria.

Differences in bacterial community composition might be also due to the lichen growth type as for instance previous studies reported that the bacterial community compositions of crustose lichens differed from those of foliose or fruticose lichens (Grube et al., 2009; Hodkinson et al., 2012). While the foliose lichens were mainly colonized by *Alphaproteobacteria*, the crustose lichen *Ophioparma* sp. was dominated by *Acidobacteria* (Hodkinson et al., 2012). Another rock-inhabiting crustose lichen *Hydropunctaria* sp. was mainly colonized by *Cyanobacteria*, *Actinobacteria*, and *Deinococcus* (Bjelland et al., 2011). But growth type on its own does not explain the predominance of certain taxa since the foliose lichen *Solorina* sp. was also dominated by *Acidobacteria* (Grube et al., 2012). This agrees with previous results of Cardinale et al. (2012b) who showed that growth types do not affect the main bacterial community structure.

## Bacteria are Spatially Structured on Lichens

Thallus sub-compartments of varying age as well as external and internal surfaces offer chemically and physiologically distinct micro-niches and facilitate the formation of various distinct bacterial communities. Based on FISH and CLSM the lichen-associated eubacteria as well as specific bacterial taxa therein were demonstrated to colonize distinct lichen thallus parts in different abundances and patterns (Cardinale et al., 2008). Confocal laser scanning microscopy of the *L. pulmonaria* surfaces showed that both the upper and the lower cortexes were evenly colonized by *Alphaproteobacteria* among other eubacteria (Cardinale et al., 2012a; Grube et al., 2015). This was also demonstrated for other dorsiventrally organized lichen thalli such as the leafy *Umbilicaria* sp. (Grube et al., 2009). In the case of the shrubby species *Cladonia* the outer cortex of the radially organized hollow thallus (podetium) was merely colonized by single cell colonies and smaller colony clusters, while the highest bacterial density examined on this lichen was found on the internal layer of the podetia forming a biofilm-like coat (Cardinale et al., 2008, 2012b). Contrarily, bacterial colonization on crustose lichens such as *Lecanora* sp. was distinctly higher in the cracks between the areoles of the thalli (Grube et al., 2009). There were also first indications for endobiotic bacteria within the cell walls of fungal hyphae (Cardinale et al., 2008). Erlacher et al. (2015) previously reported in *L. pulmonaria* endosymbiotic *Rhizobiales*, localized in varying depths of the interhyphal gelatinous matrix of the upper cortex and seldom in the interior of fungal hyphae. So far, there is no documentation of bacterial growth in other compartments of *L. pulmonaria* such as the internal thalline tissue (medulla) or the photobiont layer.

The age states in a mature lichen thallus might influence and shape bacterial community structure, which resembles the community succession found, e.g., in the apple flower microbiome (Shade et al., 2013). A recent study has shown that the vegetative propagules of *L. pulmonaria* were colonized by a more distinct bacterial community than the mature lichen thallus (Aschenbrenner et al., 2014) indicating that the community structure might change over time. In detail, only 37% of thallus-associated bacterial OTUs were shared with the vegetative propagules, conversely, shared OTUs associated with the propagules comprised 55%. While both lichen parts were mainly colonized by *Alphaproteobacteria*, the lichen thallus was additionally dominated by *Deltaproteobacteria*, whereas the juvenile vegetative propagules were also colonized in higher abundances by *Spartobacteria* and *Sphingobacteria*. Previously, Cardinale et al. (2012b) reported that older thallus parts hosted significantly higher amounts of bacteria than the younger thallus structures including a change of the predominant *Alphaproteobacteria* to other taxa such as *Actinobacteria*, *Gamma*-, and *Betaproteobacteria*. Also Mushegian et al. (2011) observed a spatial diversification of the bacterial compositions between the more diverse and consistent thallus centers (older parts) and those of the more variable and species poor edges (younger parts). Cardinale et al. (2012b) referred to this bacterial distribution patterns as anabolic centers in the growing and catabolic sinks in the senescing parts of the lichen thallus, respectively. The hypothesis of recycling nutrients in the decaying lichen parts by bacteria can be also underpinned by the presence of specific taxa known for their degradation potential. *Sphingomonas* sp., which are known to degrade organic matter and xenobiotic substances, were previously isolated from lichens sampled in Arctic and Antarctic regions (Lee et al., 2014), but also reported in other studies (Grube et al., 2009, 2012; Hodkinson et al., 2012; Aschenbrenner, 2015). However, also other genera such as *Paenibacillus* and *Streptomyces* were mentioned for their functions (e.g., chitinolytic activity) in the degradation of lichen tissues (Cardinale et al., 2006).

## Distribution and Transfer of Host-Associated Bacteria

Analyses of lichen-associated bacteria revealed differences in community composition and diversity among geographically distant habitats (Printzen et al., 2012; Aschenbrenner et al., 2014). Printzen et al. (2012) analyzed the geographic structure of lichen-associated *Alphaproteobacteria* in Antarctic regions indicating that this group is affected by environmental parameters since thalli from sub-polar habitats had more similar communities than those from extrapolar regions. Hodkinson et al. (2012) explained these large-scale geographical effects by the dispersal efficiency of the lichen hosts, where the dispersal happens on small spatial scales rather than on large-scale distances resulting in a geographic differentiation of the community composition. Aschenbrenner et al. (2014) visualized and described the bacterial colonization of lichen propagules. Their results demonstrate that at least a certain proportion of the lichen microbiome is transferred vertically via these symbiotic structures. These bacterial communities were dominated by *Alphaproteobacteria*, as was already found by Cardinale et al. (2012a). Interestingly, the bacterial consortia of the lichen propagules were more than only a subset of the parental thallus microbiome and also comprised unique species, not shared by the mature thallus. Thus, Aschenbrenner et al. (2014) suggested that the vegetative propagules are equipped with a bacterial starter community. Such bacteria colonizing juvenile structures might influence the subsequent recruitment of new bacteria (Fukami, 2010), thereby shaping the community composition. The importance of the lichen-associated bacteria during the establishment of the lichen symbiosis was already suggested (Hodkinson and Lutzoni, 2009), as the growth of stratified lichen thalli was so far only successful in cultures based on lichen fragments, which apparently include bacteria.

Although vertical transmission of lichen-associated bacteria was only shown in a single lichen species, it is very likely that this strategy of microbiome transfer is also common in other species utilizing vegetative diaspores for reproduction, and definitely in other symbioses. There are various examples reporting on a transmission of host-associated bacteria (Bright and Bulgheresi, 2010), e.g., in marine sponges (Wilkinson, 1984; Li et al., 1998). Bacteria associated with terrestrial invertebrates such as insects are known to assist in nutrient uptake and provision of essential amino acids and vitamins (Douglas, 1998; Feldhaar and Gross, 2009), but their vertical transmission strategies vary among distinct species (Sacchi et al., 1988; Attardo et al., 2008; Prado and Zucchi, 2012). In vertebrates including humans the transfer of maternal microbes to the child through natural birth and breast feeding as first inoculum was reported to be important for the baby’s health, in particular by shaping the microbiome structure with beneficial microbes (Funkhouser and Bordenstein, 2013). But also in the plant kingdom transfer of plant-associated bacteria, in particular of seeds, from the mother plant was reported (van Overbeek et al., 2011), even though it is common for higher plants to recruit their substantial rhizosphere communities from the surrounding soil (Berg and Smalla, 2009). Vertical transmission was previously shown for the oldest group of land plants, mosses, which belong together with lichens to the group of poikilohydric cryptogams; associated bacteria, especially specific *Burkholderia* strains, are transferred from the sporophyte to the gametophyte via spores (Bragina et al., 2012, 2013).

### Lichens as Bacterial Hubs

Lichens are pioneers in the colonization of hostile environments with extreme temperatures, desiccation, and high salinity, but they may also become very old, either as individuals or as associations (it is assumed that some non-glaciated sites were colonized by lichens since the tertiary). Colonized habitats include arid and semi-arid regions where bare soil can be colonized by, e.g., cryptogamic soil crusts (an association comprising soil particles, lichens, cyanobacteria, algae, fungi, and bryophytes; Beckett et al., 2008), but also more extreme regions such as deserts, where lichens are one of the few successful colonizers. In particular, their capability to become hydrated without contact to liquid water (Printzen et al., 2012) only by fog, dew or high air humidity (Beckett et al., 2008) ensures survival in these dry areas. This suggests that lichens as slow-growing and long-living host organisms might serve as bacterial hubs in these environments facilitating their survival by nutrient and water supply, offering a habitat with various micro-niches and ensuring their distribution over short distances by the dispersal strategies of the lichen host. Thereby the lichens could be important sources/reservoirs of beneficial bacterial strains for other habitats in an environment as well.

### Habitat Specificity

Host specificity for cryptogams (i.e., lichens and mosses) was already reported in previous independent studies (Grube et al., 2009; Bragina et al., 2012). However, bacterial communities were so far described almost always without a view of adjacent habitats and potential inter-microbiome relationships. Previously bacterial specificity was reported in studies of lichen thalli and their underlying rock substrate (Bjelland et al., 2011). A recent study within the doctoral thesis of Aschenbrenner (2015) focusing on this topic unraveled the specificity of the lichen-associated microbiome compared with the neighboring habitats, i.e., moss and bare bark. This comparative analysis highlighted potential habitat specialists and generalists. In this survey, members of the genus *Sphingomonas* were identified as generalists in all the three habitats, whereas members of *Mucilaginibacter* were described as potential specialists of lichens. The lung lichen frequently establishes on mosses, and the sharing of *Nostoc* strains between both cryptogams suggests a previously undescribed form of ecological facilitation that is mediated by the shared microbiome fraction (Aschenbrenner, 2015). The lung lichen takes up *Nostoc* strains during growth and incorporates them in the thallus as distinct clusters (known as internal cephalodia in the literature). As *Nostoc* is enriched on mosses rather than on bark, the growth promoting effect of nitrogen-fixing *Nostoc* apparently facilitates the efficient development of the lichen thallus, which mostly emerges from moss patches.

## The Lichen-Associated Microbiome Plays a Central Functional Role in the Lichen Holobiont

While the host-specific bacterial colonization of various lichen species was demonstrated over the past years, the roles of the bacteria remained largely unknown. This is mainly due to inherent problems to study lichens by experimental approaches (especially re-synthesis of the symbiosis in culture). Meta-omics meanwhile emerged as a set of suitable technologies to globally identify potentially beneficial contributions of the bacterial population. Recently, the *L. pulmonaria* associated microbiome was investigated with an integrated metagenomics and metaproteomics approach to screen for potential functions encoded in genomes and to verify their expression at the protein level (Grube et al., 2015), based on a previous pioneering proteomics study (Schneider et al., 2011). The results of Grube et al. (2015) provided strong evidence that the bacterial microbiome is involved in nutrient provision and degradation of older lichen thallus parts, biosynthesis of vitamins and hormones, detoxification processes, and the protection against biotic as well as abiotic stress. Additionally, the high prevalence of bacterial nitrogen fixation was confirmed with –omic data and quantitative RT-PCR. Moreover, a comparison of the whole *Lobaria*-associated metagenome with a representative set of publicly available metagenomes highlighted its uniqueness. The most closely related metagenomes were found to be those obtained from plant-associated habitats.

In particular, *Rhizobiales* (*Alphaproteobacteria*) were previously shown to be remarkably abundant in the *L. pulmonaria* microbiome mainly represented by the families: *Methylobacteriaceae*, *Bradyrhizobiaceae*, and *Rhizobiaceae*. Although they are well known for their beneficial interactions with many higher plants, less is known about their specific roles in terms of the lichens. According to Erlacher et al. (2015) functional assignments based on hierarchical SEED classification indicated an involvement of *Rhizobiales* in various beneficial functions (e.g., auxin, folate, and vitamin B12 biosynthesis). A further breakdown demonstrated that the predominant *Methylobacteriaceae* were also the most potent producers of the examined metabolites. These findings suggest the potential for various biotechnological applications of this group.

### Stress Amelioration and Pathogen Defense Functions are Supported by Metagenomic Data and Culturable Members of the Microbiome

Recently, it was shown that the *L. pulmonaria* associated microbiome includes also various bacteria with antagonistic potential (Cernava et al., 2015a). The most abundant antagonists were assigned to *Stenotrophomonas, Pseudomonas, Micrococcus*, and *Burkholderia*. These genera accounted for 67% of all identified antagonistic bacteria. Metagenomic screening revealed the presence of genes involved in the biosynthesis of stress-reducing metabolites. Complementary high-performance liquid chromatography-mass spectrometry (HPLC-MS) analyses enabled the detection of *Stenotrophomonas*-produced spermidine which is known to reduce desiccation- and high-salinity-induced stress in plants. It was also tested if these protective effects can be transferred to non-lichen hosts such as primed tomato (*Solanum lycopersicum*) seeds. Results indicated a significant increase in the root and stem lengths under water-limited conditions. The application of lichen-associated bacteria in plant protection and growth promotion may prove to be a useful alternative to conventional approaches. However, further studies are required to evaluate the host range and to elucidate the overall applicability (Cernava et al., 2015a).

Furthermore, volatile organic compounds (VOCs) profiles from bacterial isolates showed that lichen-associated bacteria are emitting a broad range of volatile substances. These molecules are most likely involved in various interactions (e.g., communication between microorganisms and the host) and might also increase the overall resistance against various pathogens (Cernava et al., 2015b).

### The Microbiome Provides Complementary Detoxification Mechanisms

Besides the evidence for mechanisms conferring enhanced resistance against biotic as well as abiotic stress, the microbiome provided a first evidence for the involvement in the detoxification of inorganic substances (e.g., As, Cu, Zn), the detailed mechanisms remaining unknown. A deeper insight into these beneficial contributions was possible with samples exposed to elevated arsenic concentration (Cernava, 2015). Metagenomic analyses revealed that the overall microbial community structures from different lichens were similar, irrespective of the arsenic concentrations at the sampling locations, whereas the spectrum of functions related to arsenic metabolism was extended. These functions include bioconversion mechanisms that are involved in the methylation of inorganic arsenic and consequently generate less toxic substances. Furthermore, the abundance of numerous detoxification related genes was enhanced in arsenic-polluted samples. Supplementary qPCR approaches have shown that the *ars*M gene copy number is not strictly related to the determined arsenic concentrations. Additionally, a culture collection of bacterial isolates obtained from three lichen species was screened for the *ars*M gene. Detected carriers of *ars*M were later identified as members of the genera *Leifsonia, Micrococcus, Pedobacter, Staphylococcus*, and *Streptomyces*. The overall results underscored the important role of the microbiome in host protection and they provided more detailed insights into the taxonomic structure of involved microorganisms.

## Bacterial Microbiome Assembly on a Symbiotic Fungal Structure

**FIGURE 1:**
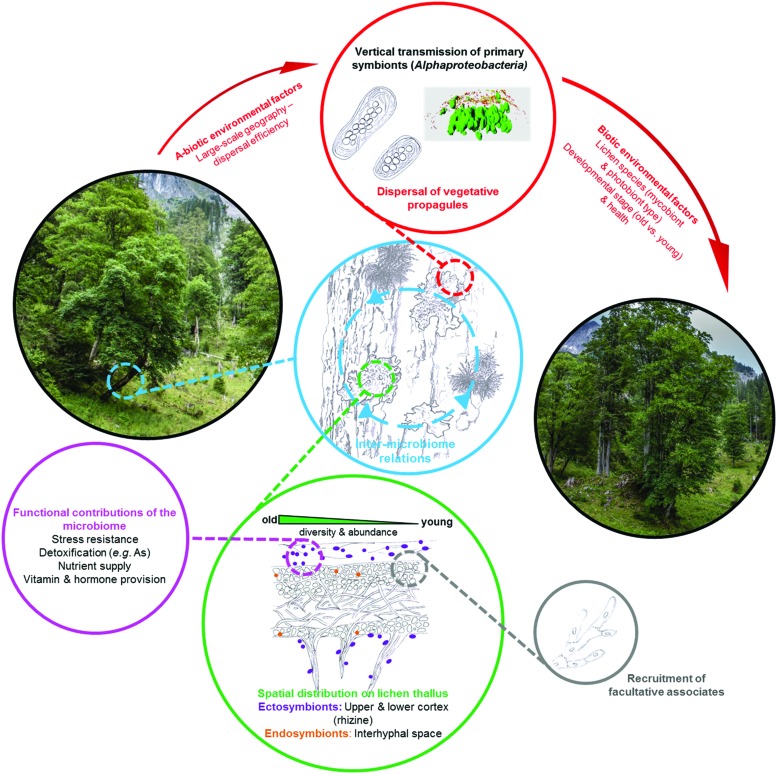
**A holistic view of the lichen microbiome diversity and identified functions in the environmental context**. Lichen-associated bacterial communities were shown to share substantial fractions of identified taxa with adjacent microhabitats (blue circle). This suggests a dynamic acquisition and exchange of beneficial species. Specific proportions of the microbiome are vertically transmitted to the next generation and used for the establishment of novel populations (red circle and arrows). Highly diverse bacterial populations primarily colonize outer lichen layers, but some can also enter the inter-hyphal matrix (green circle). External factors provide a shared microbial ‘core assembly’ of the habitat, but host-specific factors (gray circle) determine the lichen-specific bacterial community, which contributes a variety of beneficial functions for the host symbiosis (purple circle).

The lichen thallus with its various micro-niches represents a miniature ecosystem for microorganisms. While lichen-associated bacteria were previously neglected and often recognized as contamination of lichen thalli, recent research considers them – with increasing evidence – as important and crucial component of the lichen meta-organism. By their microbiomes lichens are ecologically linked with their surrounding environment (**Figure 1**). Even though a fraction of their microbiome can be transmitted by local dispersal of vegetative propagules, further recruitment of strains occurs from the local resources in the environment. This finally leads to a specific community structure of mature lichen thalli, which shares a core microbiome over larger distance (Aschenbrenner et al., 2014). Lichen thalli, already present on Earth since the lower Devonian, and representing the most complex vegetative structures in the fungal kingdom, may have evolved as bacterial enrichment structures. The exposed surfaces of lichens are ideally suited to benefit from functions of adapted and enriched bacteria, or from degradation of spurious non-adapted bacteria caught from the environment. The bacterial harvest may readily be dissipated to the symbiotic corporates via the fungal textures. It is this new perspective of the lichen symbiosis, which offers a wide range of new research questions in the near future.

## Conclusions – Lichens as a Case Model to Understand Multi-Species Symbioses

Undoubtedly, there exist other cases of symbioses involving multiple organismal groups in terrestrial ecosystems. Similar to lichens, these were originally recognized as dual eukaryotic partnerships, but later shown to involve specific bacterial associations as well (e.g., fungi/leaf-cutter ants, Little and Currie, 2007; mycorrhiza, Garbaye, 1994). Modern tools now overcome the difficulties to re-establish complex symbioses under axenic laboratory conditions, and moreover, they allow us to precisely study symbioses in their environmental context. We consider lichens as ideal research objects for this purpose, because in contrast to many other symbiotic systems, they have an unsurpassed ecological range in general, but with rather specific adaptation of each species to their ecological niches. It will thus clearly be a novel and highly interesting theme in symbiotic research to establish the role of the microbiome in ecological adaptation and evolution of the lichen multi-species symbiosis.

## Author Contributions

IA, TC, GB, and MG wrote the manuscript. IA and TC contributed with results from their Ph. D. studies. GB and MG complemented the manuscript with profound experience in the fields of microbiome and lichen research.

## Conflict of Interest Statement

The authors declare that the research was conducted in the absence of any commercial or financial relationships that could be construed as a potential conflict of interest.
